# Editorial: Biodegradable polymeric materials in tissue engineering and their application in drug delivery

**DOI:** 10.3389/fbioe.2023.1296119

**Published:** 2023-09-29

**Authors:** Sudarshan Singh, Chuda Chittasupho, Bhupendra G. Prajapati, Arvind Singh Chandel

**Affiliations:** ^1^ Department of Pharmaceutical Sciences, Faculty of Pharmacy, Chiang Mai University, Chiang Mai, Thailand; ^2^ Office of Research Administration, Chiang Mai University, Chiang Mai, Thailand; ^3^ Shree S. K. Patel College of Pharmaceutical Education and Research, Ganpat University, Kherva, India; ^4^ Center for Disease Biology and Integrative Medicine, Faculty of Medicine, The University of Tokyo, Tokyo, Japan

**Keywords:** biopolymer, tissue engineering, three dimensional printing (3D printing), scaffold, composite

Polymeric materials obtained from petroleum resources are non-biodegradable. Defying degradation, they damage the environment as a result of their ending up in the landfills. Natural and synthesized biodegradable polymeric materials have received increasing interest owing to the difficulty in biocompatibility and reproducibility, compared to non-biodegradable polymeric materials. Moreover, the modification of natural polymeric materials or materials via chemical, microbiological, enzyme-mediated, and chemo-enzymatic synthesis, a compressive range of variegated biodegradable polymeric materials can be replaced.

In recent years, significant advancement has been made in drug delivery system using both natural and synthetic polymers. Polymers are a critical material class due to their wide availability, range of properties and high tuneability. These polymeric materials have inherent flexibility in that they can be synthesized and modified to provide versatile properties to meet the desired controlled drug release profile with biocompatibility. Polymers are commonly used by pharmaceutical manufacturer in the fabrication of tablets, implants, microspheres, nanoparticles, drug-eluting stents, *in situ* forming gels, and polymeric scaffolds for tissue engineering to achieve the goal of drug delivery system. Increasingly, researchers are using data science and polymer informatics to design new materials and understand their structural property relationships. Material performance is highly linked to strength, porosity, particle size, amorphous nature, biocompatibility, and dissolutions performance.

Biodegradable biomaterials are categorized as natural and synthetic-based on their sources and whether they are composed of naturally occurring extracellular matrix. Natural polymeric biomaterials include proteins (collagen, fibrin, silk, *etc.*), and polysaccharides (chitin/chitosan, alginate, hyaluronic acid, lignin, *etc.*). Whereas a family of native polysters-polyhydroxyalkanoates has been recognized as natural biodegradable biomaterials and, more recently, sundew adhesives, and ivy nanoparticles have garnered more attention for their ability to develop effective nanocomposite adhesives and for their potential use as nano-carriers in various drug delivery. Moreover, these biomaterials have been significantly explored in customized three/four dimensional bioprinting for the management of otolaryngology (Vyas et al.).

Chitin and chitosan are a type of polysaccharides that are found in abundance in various marine sources. Chitin is a polymer composed of β,1-4-linked *N-*acetyl glucosamine units and it is a complex biopolymer. Chitin present in the exoskeletons of arthropods as well as crustaceans such as crabs, lobsters, and shrimps, and it is water-insoluble and is often transformed into carboxymethyl chitosan and other chitosan derivatives. Chitin extracted from lobster processing waste is used in variety of ways in food, healthcare, agriculture, pharmaceuticals, and biomedical fields. On the other hand, chitosan is a linear polymer derived from chitin via the deacetylation process, which is made up of both *N-*acetyl glucosamine and D-glucosamine residues. Chitosan contains amine groups that are pH sensitive, making it neutral in the alkaline pH and positively charged in the acidic pH. Chitosan have been extensively explored polymeric biomaterials in fabrication of wound dressing, beads, microcapsules, microspheres, nanoparticles, food packaging, smart/intelligent food spoilage sensing composite, and scaffolds for skin tissue engineering. Furthermore, grafting of polymeric materials with other active polymers or bioactive compounds provides possibilities of developing a biomaterial with new opportunities and improved therapeutics efficacy (Purohit et al.). Chitin, chitosan, and chito-oligosaccharides are well known for endless grafting possibilities for multifarious applications (Mohite et al.).

Polyethylene glycol, which is an important hydrophilic polymer with significant biocompatibility, non-immunogenicity, and anti-protein adsorption makes it choices of polymeric materials for use in wide variety of biomedical applications. Although polyethylene glycol has an active hydroxyl end that can combine with a variety of drug active molecules to form a drug delivery system, the polyethylene glycol with two hydroxyl end groups is most commonly used as forming functional hydrogels with other monomer macromolecules or particles by photopolymerization. This polyethylene glycol-based hydrogel has been significantly explored to improve bone repair efficacy (Sun et al.).

In conclusion polymeric materials play a crucial role in tissue engineering, a field that combine principles from materials science, biology, and engineering to fabricate functional replacements for damaged or diseased tissues. Furthermore, polymeric materials serve as indispensable cornerstones in the field of tissue engineering, offering a versatile platform to develop scaffolds that support and guide tissue and bone regeneration. Their biocompatibility, biodegradability, and ability to be tailored for specific applications make them invaluable tools in the quest to develop functional replacements for damaged or diseased tissues. Through careful selection and engineering, these polymers can mimic the mechanical and biochemical properties of native tissues, fostering an environment conducive to cellular growth, differentiation, and tissue repair. As researchers continue to innovate and refine polymeric materials, we can anticipate even greater strides in the advancement of tissue engineering, offering hope for improved treatments and outcomes for patients facing a wide array of medical challenges.

